# Automated Segmented-Flow Analysis – NMR with a Novel Fluoropolymer Flow Cell for High-Throughput Screening

**DOI:** 10.1021/acs.analchem.2c03038

**Published:** 2022-10-27

**Authors:** Bert Wouters, Paul Miggiels, Roland Bezemer, Elwin A.W. van der Cruijsen, Erik van Leeuwen, John Gauvin, Klaartje Houben, Karthick Babu Sai Sankar Gupta, Paul Zuijdwijk, Amy Harms, Adriana Carvalho de Souza, Thomas Hankemeier

**Affiliations:** † Metabolomics and Analytics Centre, Leiden Academic Centre for Drug Research, Leiden University, Einsteinweg 55, 2333 CCLeiden, The Netherlands; § DSM Biotechnology Center, Alexander Fleminglaan 1, 2613 AXDelft, The Netherlands; ∥ Leiden Institute of Chemistry, Leiden University, Einsteinweg 55, 2333 CCLeiden, The Netherlands

## Abstract

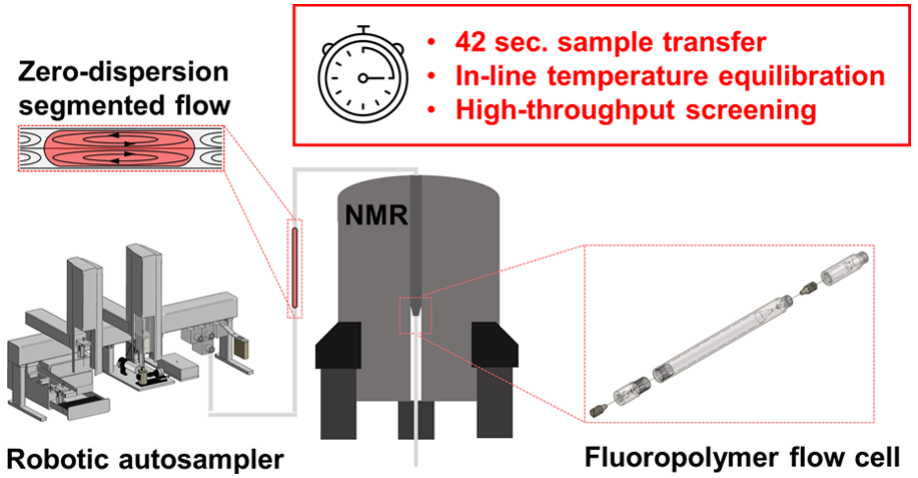

High-throughput analysis in fields such as industrial biotechnology, combinatorial chemistry, and life sciences is becoming increasingly important. Nuclear magnetic resonance (NMR) spectroscopy is a powerful technique providing exhaustive molecular information on complex samples. Flow NMR in particular is a cost- and time-efficient method for large screenings. In this study, we have developed a novel 3.0 mm inner diameter polychlorotrifluoroethylene (PCTFE) flow cell for a segmented-flow analysis (SFA) – NMR automated platform. The platform uses FC-72 fluorinated oil and fluoropolymer components to achieve a fully fluorinated flow path. Samples were repeatably transferred from 96-deepwell plates to the flow cell by displacing a fixed volume of oil, with a transfer time of 42 s. ^1^H spectra were acquired fully automated with 500 and 600 MHz NMR spectrometers. The spectral performance of the novel PCTFE cell was equal to that of commercial glass cells. Peak area repeatability was excellent with a relative standard deviation of 0.1–0.5% for standard samples, and carryover was below 0.2% without intermediate washing. The sample temperature was conditioned by using a thermostated transfer line in order to reduce the equilibration time in the probe and increase the throughput. Finally, analysis of urine samples demonstrated the applicability of this platform for screening complex matrices.

## 1 Introduction

The robust analysis of vast numbers of samples is necessary for applications such as strain selection or process monitoring for industrial biotechnology,^1^ library screening in pharmaceutical development^2^ or combinatorial chemistry,^3^ and biomarker and metabolite discovery in biofluids.^4−6^ Analytical challenges encountered have been a major driver for technology advancements toward faster and cheaper analysis with at the same time better biochemical coverage.^7^ In this regard, liquid chromatography–mass spectrometry (LC-MS) and nuclear magnetic resonance (NMR) spectroscopy are well-established and complementary analytical tools.^8−10^

NMR is a powerful tool for direct quantification, structural elucidation, and coverage. Conventional NMR with glass tubes and acquisition times of minutes to hours provides extensive sample information at low throughput. Alternatively, flow NMR is ideally suited for fingerprinting and screening applications^2^ where throughput is prioritized over depth of information. In flow NMR, the sample is transferred into a flow cell inside the NMR coil. This technique, dating back to the 1950s,^11^ has found its use in various applications such as reaction monitoring,^12−15^ compound identification,^16^ and fragment-based screening.^17−19^ It was also demonstrated for high throughput industrial screening, for instance, analyzing 11520 samples with a cycle time of only 60 s.^20^ Flow NMR has several key benefits for screening purposes. First, the fixed flow cell provides a constant environment for the samples. As a result, the magnetic homogeneity is (near-)equal for all samples of similar composition, and no or minor adjustments are needed between samples.^20,21^ Second, sample transfer can be fast, in the order of 30 s, and the sample temperature can be conditioned during transfer to shorten the equilibration time inside the probe.^20^ An additional gain in throughput could be achieved via parallel NMR detection using 2- and 4-microcoil flow multiplex NMR probes.^19,22^ Third, the cost of glass tubes for a screening of 10000 samples is significant and filling the tubes is a laborious task. Although automated solutions are available for glass tubes, commercial liquid handlers for flow NMR can readily work with standard plate formats.

Flow NMR poses two design challenges, namely, the design and material of the flow cell and the sample transfer into the cell. A plethora of flow cell materials and designs have been reported including glass-like materials,^12,23,24^ nonferromagnetic metals,^25−27^ and polymeric materials.^18,28−31^ Likewise, various strategies have been explored for transferring the sample from the autosampler into the flow cell,^32^ including direct injection (DI-NMR),^33,34^ flow-injection analysis (FIA-NMR),^17,34,35^ and segmented-flow analysis (SFA-NMR).^36^ In comparison, SFA-NMR is advantageous in minimizing sample consumption, sample dispersion, and carryover. Often air or nitrogen is used as carrier phase, though these are not ideal, as the compressibility and thermal expansion of gases can compromise positioning accuracy or induce undesired mixing. Moreover, the volume magnetic susceptibility of air differs greatly from common solvents and the copper coil,^37^ and the presence of air bubbles in or near the RF coil can significantly distort spectral quality.^38,39^ Alternatively, fluorinated oils provide several advantages for SFA-NMR: (*i*) the volume magnetic susceptibility is similar to that of copper, thus, less sample overfill (fill factor, ) can be used without degradation of the line shape,^38,39^ (*ii*) fluorinated oils are extraordinarily nonpolar and unfavorable for partitioning of organic solvents and dissolved analytes,^40^ and (*iii*) in combination with fluoropolymer tubing, the oil forms a film around the sample segments due to preferential wetting behavior, thereby greatly reducing the risk of surface fouling.^3^ The difference in preferential wetting can also be utilized to separate the sample and oil phase after analysis to recover the oil.^41,42^ Despite these advantages, the application of SFA-NMR with fluorinated oil, especially in combination with fluoropolymer flow paths, so far has only been reported for microcoil-NMR^3,43,44^ and a 1.0 mm ID Teflon flow tube.^45^ The combination of fluorinated oil with a fluoropolymer flow path and a conventional-sized fluorinated flow cell could be an ideal solution for SFA-NMR for screening purposes.

In this study, we present an automated SFA-NMR system using FC-72 fluorocarbon oil and a fully fluorinated flow path. This includes a novel 3.0 mm inner diameter PCTFE flow cell made using thermal compression molding. First, the flow cell and system performance were evaluated on spectral quality, sample-to-sample carryover, and repeatability with aqueous standards, and compared to the commercial Bruker BEST-NMR system with air segmentation and a glass flow cell. Second, the sample cycle time was reduced by preconditioning the sample temperature during sample transfer. Finally, the applicability of our system was demonstrated by the analysis of urine samples.

## 2 Methods

### Chemicals and Consumables

All water referred to in this article was deionized water (Milli-Q Integral). FC-72 oil was purchased from 3 M (Zwijndrecht, Belgium). Deuterium oxide (D_2_O, 99.9%) was purchased from Cambridge Isotope Laboratories (DLM-4). Sucrose (BioXtra, ≥99.5%), l-alanine (99.5%), glucose (99.5%), and 3-(trimethylsilyl)-1-propanesulfonic acid-d_6_ sodium salt (DSS-d_6_) were purchased from Sigma-Aldrich. Trisodium citrate dihydrate (Ph. Eur., BP, USP) was purchased from VWR Chemicals. Acetic acid (100%) and maleic acid (Msynth plus) were purchased from Merck. Formic acid (99%) and lactic acid (99%) were obtained from Acros Organics, and citric acid (99.5%) was purchased from Carl Roth. Fluorinated ethylene propylene (FEP; 1/32” OD, 254 μm ID), perfluoroalkoxy (PFA) tubing (1/8”, 1/16” OD, 508 μm ID), and polyether ether ketone (PEEK) tubing (1/32” OD, 380 μm ID) were obtained from BGB Analytik (Harderwijk, The Netherlands). One-piece PEEK 1/32” connectors (Mengel Engineering, Virum, Denmark), of which the hex nut was removed, were used for fluidic connections at the flow cell.

### Standard Samples

The 58 mM sucrose sample solutions consisted of 1985 mg sucrose and 2.5 mg DSS-d_6_ dissolved in 100 mL deuterium oxide (D_2_O). Seventeen mM maleic acid sample solutions consisted of 197 mg maleic acid and 2.5 mg DSS-d_6_ dissolved in 100 mL D_2_O, pH adjusted to 6.40 with NaOH. The 17 mM citrate solution consisted of 439 mg trisodium citrate and 2.5 mg DSS, dissolved in 100 mL D_2_O. Blank solutions consisted of 2.5 mg DSS-d_6_ dissolved in 100 mL D_2_O.

### Urine Sample Preparation

Urine was collected from six healthy volunteers (aged 25–40 yrs) and stored at −80 °C for several weeks. Spiking solutions were prepared in water; a glucose solution of approximately 16 mM, and an acidic standards solution containing approximately 160 mM acetic acid, 400 mM citric acid, 80 mM formic acid, 160 mM lactic acid, and 96 mM alanine. The stored urine was thawed at room temperature, pooled, and thoroughly mixed. Aliquots of 50 mL were centrifugated for 5 min at 2000 rcf. Next, 100 μL of Bruker VERBR urine buffer (Bruker Biospin GmbH, Rheinstetten, Germany) was pipetted into 96-deepwell plates and 900 μL of urine, or water for blanks, was added. For the spiked samples, 10 μL of glucose spiking solution or 25 μL of the acidic standards spiking solutions was added. The plates were heat-sealed with a pierceable aluminum seal and shaken for 30 s on a plate shaker, and stored refrigerated for several days until analysis.

### Fabrication of PCTFE Flow Cells

**Figure 1:**
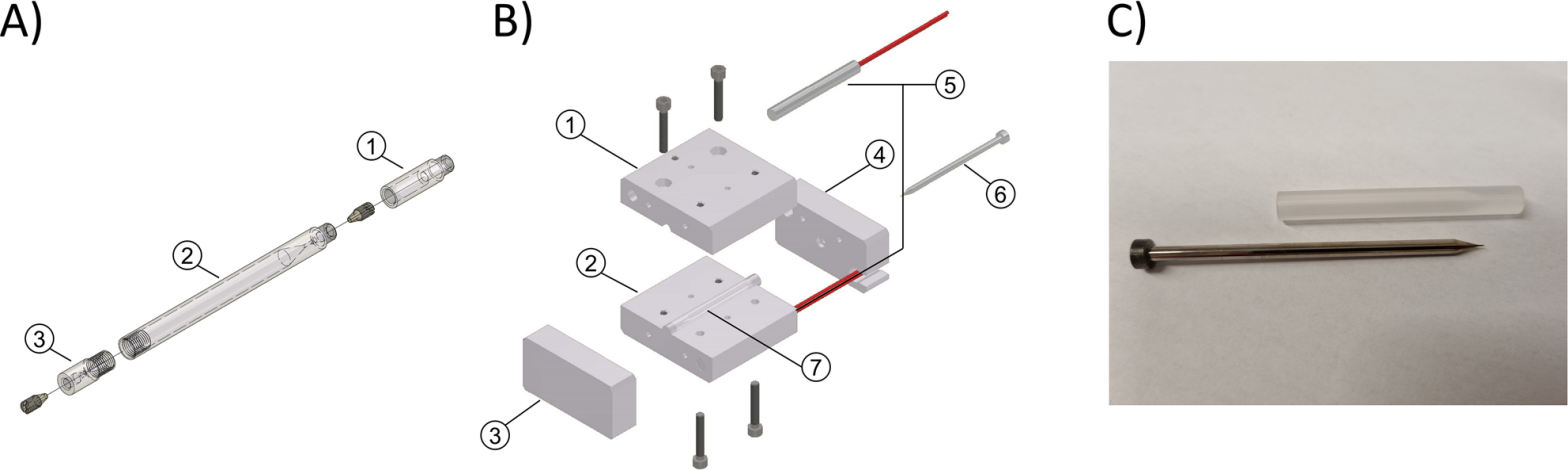
(A) Exploded view of the PCTFE flow cell with the inlet (1), main body (2), and outlet (3); (B) Compression molding setup with its parts; 1–4: main body; 5: heating rods; 6: steel core mold; 7: flow cell blank. (C) Photograph of the core mold and a partially assembled PCTFE flow cell body after compression molding.

NMR flow cells were fabricated by the Fine Mechanical Department at Leiden University. All parts were machined from 10 mm diameter polychlorofluoroethylene (PCTFE) rods (100% PCTFE, ERIKS, Alkmaar, The Netherlands). The flow cell consists of three parts (Figure 1A); an inlet (1), the main body (2), and an outlet (3). The in- and outlet were fabricated by drilling and turning on a Schaublin 125-CF high precision screw-cutting lathe (Schaublin A.G., Bélivard, Switzerland). The main body was first dimensioned to 5.0 mm outer diameter, and the initial inner geometry was created on the lathe. The conical transitions were smoothed with a 30° tipped endmill and a custom reamer tool. At this stage, the inner bore was left at 3.2 mm for further processing.

After initial fabrication, the inner surface of the flow chamber was smoothed with thermal compression molding around a core mold. The compression chamber and parts are illustrated in Figure 1B. The main body (1–4) was assembled with guiding pins to ensure alignment of all parts and screwed tightly together. It was fitted with two 200-W heating rods and a k-type thermocouple, connected to an Omega CN7853 controller (Omega Engineering Limited, Manchester, United Kingdom) to control the process temperature. A hardened steel pin was used as core mold (5), which was machined to the final dimension on the lathe and finely polished to a mirror-smooth finish. The core mold covered the flow chamber of the flow cell, the conical transition, and the inlet. The procedure was as follows: the core mold was inserted into the flow cell blank; these parts were inserted into the molding chamber; the entire assembly was placed in a bench vise and clamped hand-tight. The core mold had a flat head that protruded from the chamber, pressing the mold axially into the PCTFE blank when tightening the vise. Next, the assembly was heated to 150 °C for 15 min, the temperature was further increased to 210 °C and maintained for 30 min, after which the power was turned off and the assembly was left to cool for 30–45 min. Finally, the flow cell was removed from the assembly and the core mold was extracted using an axial extraction tool. The core mold and resulting flow cell body are shown in Figure 1C. As finishing steps, the main body was turned to 4.0 mm outer diameter and the friction fit on the outlet was dimensioned on the lathe.

### Flow NMR Platform

A CTC PAL3 Dual-Head Robot RTC/RSI 160 cm robotic autosampler (CTC Analytics AG, Zwingen, Switzerland) was fluidically coupled to a Bruker spectrometers either an Avance III HD 500 MHz UltraShield Plus (*B*_1_ = 500.2299 MHz) or an Avance III HD 600 MHz Ascend (Bruker Biospin GmbH, Rheinstetten, Germany). Both spectrometers were equipped with a 5.0 mm helium-cooled CryoProbe head (CP TCI 500S2 H–C/N-D-05 Z). The flow cell was positioned in the probe using the umbilical accessory from the Bruker InsightMR system. The original adapter of the umbilical was replaced by a custom polyoxymethylene (POM) adapter that features a single through-hole for the transfer tubing and a lengthened stem with a threaded end to receive the flow cell. The stem length was designed such that center of the flow cell was at the midplane of the RF coil. The outlet of the flow cell was connected to a piece of 1/32” OD, 380 μm ID PEEK tubing that exits through the cryoprobe at the bottom of the spectrometer, thus creating a flow-through system. The water of the umbilical accessory was thermostated with a Julabo F25-HE circulator (Julabo GmbH, Seelbach, Germany).

Samples were taken from a 96-deepwell plate and injected into a custom 6-port, 2-position CTFE/PTFE injection valve (VICI AG, Schenkon, Switzerland) fitted with a 290 μL PFA loop (1/16” OD, 508 μm ID). A total of 330 μL of the sample was loaded to ensure proper loop filling, and segments were positioned in the flow cell by displacement of a fixed volume of FC-72 oil with a VICI M50HP pump (VICI, Schenkon, Switzerland) for stop-flow analysis. The tubing was PFA (1/16” OD, 508 μm ID) from the pump to the valve and FEP (1/32” OD, 254 μm ID) from the valve to the flow cell. An industrial pressure transducer (type M3021, 0–35 bar, TE Connectivity GmbH, Steinach, Switzerland) was placed between the pump and the valve to monitor the pressure at the inlet of the sample transfer system during experiments. A 5 psi (35 kPa) backpressure regulator (IDEX, Erlangen, Germany) was connected to the outlet tubing.

Reference spectra were acquired using the Bruker Efficient Sample Transfer system (BEST-NMR) which uses a Gilson 215 liquid handler coupled to a Bruker Avance III 500 MHz UltraShield (*B*_1_ = 500.1299 MHz) spectrometer (Bruker Biospin GmbH, Rheinstetten, Germany). This system uses air as immiscible carrier, PEEK transfer tubing, and a glass flow cell with 120 μL active volume (Bruker part no. H13792). The sample sequence was (*i*) 200 μL of air gap, (*ii*) 200 μL of wash solvent (D_2_O), (*iii*) 200 μL of air gap, (*iv*) 350 μL of sample, (*v*) 200 μL of air gap, and (*vi*) push solvent (D_2_O) to center the sample in the flow cell, resulting in a fill factor of 2.92. Samples were dispensed at a rate of 4 mL·min^–1^.

### Automation and Integration

The CTC PAL autosampler was programmed and controlled via PAL Sample Control 2.50 (CTC Analytics AG, Zwingen, Switzerland). Timing of the sample injection, sample transfer, and NMR acquisition were orchestrated by an in-house developed Python interface. PAL Sample Control acted as the master and all sample parameters, such as transfer volume and NMR parameters, were entered in the sample list. This sample data was shared as a text file with the Python interface, which in turn submitted the experiment just-in-time to the IconNMR queue via the external setup file automation method over the internal network. The IconNMR HyStar LCNMR protocol via an RS-232 connection was used to time the acquisition with the Python interface. An Arduino Mega 2560 R3 (Arduino, Italy) was programmed to convert serial commands to contact-closure signals and vice versa to synchronize the sample injections between the autosampler and the Python interface. When the sample was loaded in the injection loop, the Python interface started the pump (connected via RS-485) to displace a specified volume. After displacing the volume, the interface communicated to IconNMR to start the acquisition. While the acquisition was in progress, the autosampler prepared for the next injection. The next injection was injected directly after the acquisition had finished to maximize the throughput.

### NMR Acquisition and Processing

Performance of the system was evaluated using standard solutions. ^1^H s^11^ pectra were recorded with standard pulse program (zgcppr) with following parameters: 32 scans, 2 dummy scans, 64k data points, 21.0 ppm spectral width, 1.2 s relaxation delay (d1), 8 μs 90° pulse, 3.12 s. acquisition time, 5 Hz water suppression, and fixed receiver gain (rg) of 32. Spectra were processed and analyzed using Topspin 4.0.1 (Bruker). Before Fourier Transformation (FT), exponential multiplication (EM) apodization with a line-broadening factor of 0.3 Hz was used unless specified otherwise. Spectral phasing was applied and spectra were aligned to DSS-*d*_6_ at 0 ppm. Auto baseline correction was applied on the full spectrum width. Additional third-order polynomial baseline correction for selected regions was applied if needed. Peak characteristics were exported as plaintext to calculate repeatability. Carryover was determined by peak area, normalized to DSS-d_6_, using the following chemical shift ranges (in ppm): sucrose singlet 3.6–3.7, doublet 5.3–5.5; maleic acid: 5.95–6.1; citric acid 2.3–2.9. All presented figures were processed in Mnova 14.0.0 (Mestrelab Research S. L., Santiago de Compostela, Spain). Before Fourier Transformation (FT), data sets were zero-filled to 128k data points. After FT, spectra were baselined in selected regions using the Whittaker Smoother method, phased, and aligned by shifting the DDS signal to zero.

### Urine Analysis

Spectra for urine samples were recorded on the 600 MHz spectrometer with a Cryoprobe, using the default “PROF_1H” experiment with “noesygppr1d” pulse program and following changed parameters: 32 scans (64 for blanks), 64k data points, 2.595 s acquisition time, fixed receiver gain (rg) of 64, 25 Hz water suppression, and a mixing time (d8) of 10 ms. Locking, tuning and matching, and shimming were done on a urine sample prior to running the sequences and then disabled for the entire sequence. Spectra were processed and analyzed as above, but spectra were aligned to TSP at 0 ppm. Carryover of the spiked analytes was calculated from the peak areas, normalized to TSP. All presented figures were processed in Mnova 14.0.0 with zero-filling and FT as above. After FT, spectra were baselined in selected regions using a polynomial fourth order fit, phased, and aligned by shifting the TSP signal to zero. NMR signals from the urine samples were tentatively assigned based on their chemical shift.

## 3 Results and Discussion

### Fluorinated SFA-NMR Platform

**Figure 2:**
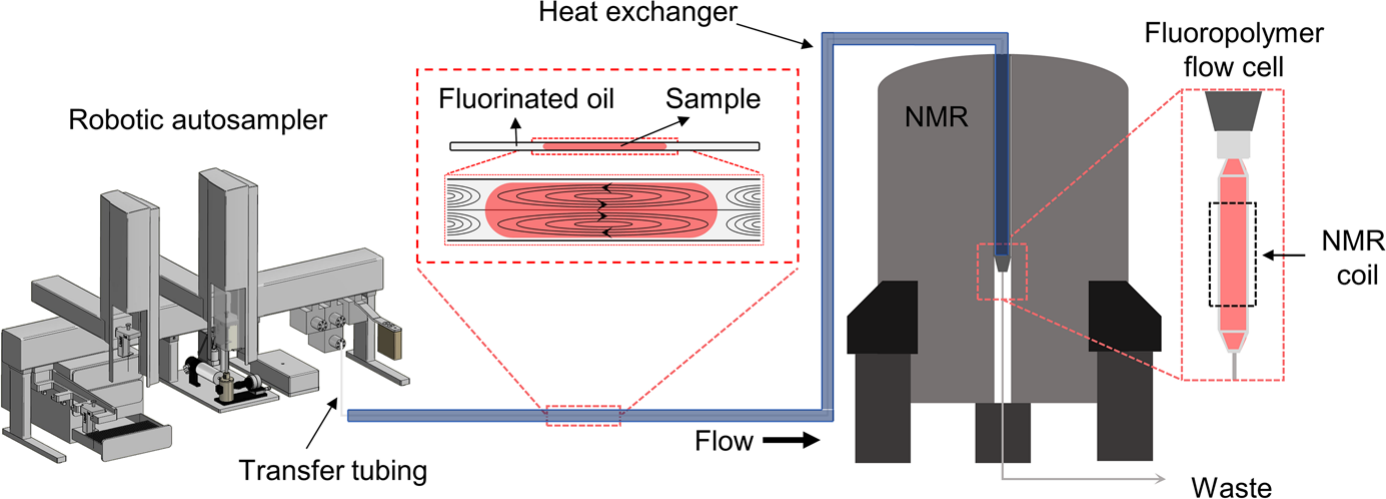
Schematic overview of the segmented-flow analysis – NMR platform with fluorinated oil and a fluoropolymer flow cell. From left to right: robotic autosampler, fluoropolymer transfer tubing in coaxial heat exchanger, and an NMR spectrometer fitted with a flow cryoprobe and an in-house developed fluoropolymer flow cell. Insets in the middle and right show segmented flow in the transfer capillary and the flow cell and NMR coil, respectively.

The developed platform (Figure 2) uses narrow-ID FEP transfer capillaries to minimize sample transfer times and a custom 3.0 mm ID PCTFE flow cell for maximum detection volume. A fully fluorinated flow path was achieved with a CTFE/PCTFE injection valve and PFA loop. FC-72 fluorocarbon oil was selected as the carrier oil for several key properties; it is immiscible with water and most common organic solvents,^40^ is chemically inert, and has a low boiling point of around 56 °C which means it could be distilled for reuse. Moreover, it has good magnetic susceptibility matching to copper and water^37,39^ and shows minimal background signal in ^1^H NMR (Figure S1), as it is fully fluorinated.

### Flow Cell Development

PCTFE was chosen as flow cell material for its chemical inertness and excellent dimensional stability. Its physical properties are highly suited for precision-machining methods thermoforming approaches,^46,47^ which allows to fabricate rigid, thin-walled flow cells with high surface quality. Moreover, its polymeric structure has no protons and the magnetic susceptibility is close to that of copper^37^ (reference spectra in Figure S2). Other fluoropolymers were briefly tested but abandoned due to disturbances in the baseline caused by protons in the polymeric structure (data not included).

The flow cell, as seen in Figure 1A, consists of three parts: (1) connection to the umbilical adapter and the transfer capillary; (2) main body with conical transition from the 250-μm inlet to the 3.0 mm internal diameter × 33 mm length section, and (3) outlet with similar inversed conical transition to connect the outlet waste tubing. A detailed technical drawing of the flow cell is provided in the Supporting Information. The inlet part (1) is attached to the main body (2) with a threaded connection, and the outlet (3) is secured with a ridge-and-groove press fitting to ensure a mechanically robust and leak-free assembly of the flow cell. The one-piece PEEK fittings ensure reversible, pressure-resistant, minimal-dead-volume connections for the in- and outlet tubing. Before first use, the flow cells were tested to withstand an operating pressure of 2 MPa for at least 30 min. Subsequently, the flow cells were routinely used at 0.5–1 MPa. In this design, the 33 mm long sample chamber has a volume of approximately 233 μL, has a constant wall thickness of 0.5 mm, and extends beyond the 22 mm long RF coils. The active sample volume (*V*_s_ = *L*_RF_π(*R*_s_)^2^) is approximately 156 μL, resulting in a fill factor of 1.86. The smooth conical transitions prevent “jetting in” of the sample or stagnant zones at the in-and outlet.^12,16,48,49^

The compression molding step was added to the manufacturing process to smoothen the inner flow cell surface from markings left by the abrasive machining methods. Microscale undulations can lead to inhomogeneities in sample distribution and consequently in the magnetic field. Compression molding is based on the crystalline properties of PCTFE and the possibility to superficially deform the substrate below its melting point. Heating the material above its glass temperature (*T*_g_ = 50–55 °C) but below the melt temperature (*T*_m_ = 210–215 °C) enables limited mobility of the polymer chains and thereby small deformations of the surface without degrading the polymer or changing the geometry.^47^ By pressing the core mold into the heated substrate, the surface quality of the mold is then transferred to the inner surfaces of the flow cell. The subsequent slow cooling allows material to set in its new shape while minimizing internal stresses that could cause fractures.

### Sample Transfer

The sample is positioned in the flow cell by displacement of a fixed volume of FC-72 oil. In principle, the transfer volume is given by . The transfer volume was further finetuned using 1D B_o_ field homogeneity to assess the filling quality. Effective filling could be achieved with a large margin around the estimate and a transfer volume of 565 μL was selected to symmetrically position the sample around the midplane of the RF coil, resulting in a sample transfer time of 42 s at a flow rate of 0.8 mL/min and maximum system pressure of 0.8–1.0 MPa. In this case the pressure rating of the injection valve (250 psi or 1.72 MPa) was the limiting factor for further increasing the flow rate. The transfer time could be shortened by placing the autosampler closer to the NMR, depending on the layout of the system. The piston-driven positive displacement pump in combination with a backpressure regulator proved capable of accurately and repeatably positioning the sample in the flow cell without the need for additional positional feedback and maintaining position for several hours.

### Spectral Quality, Repeatability, and Sample-to-Sample Carryover

**Figure 3:**
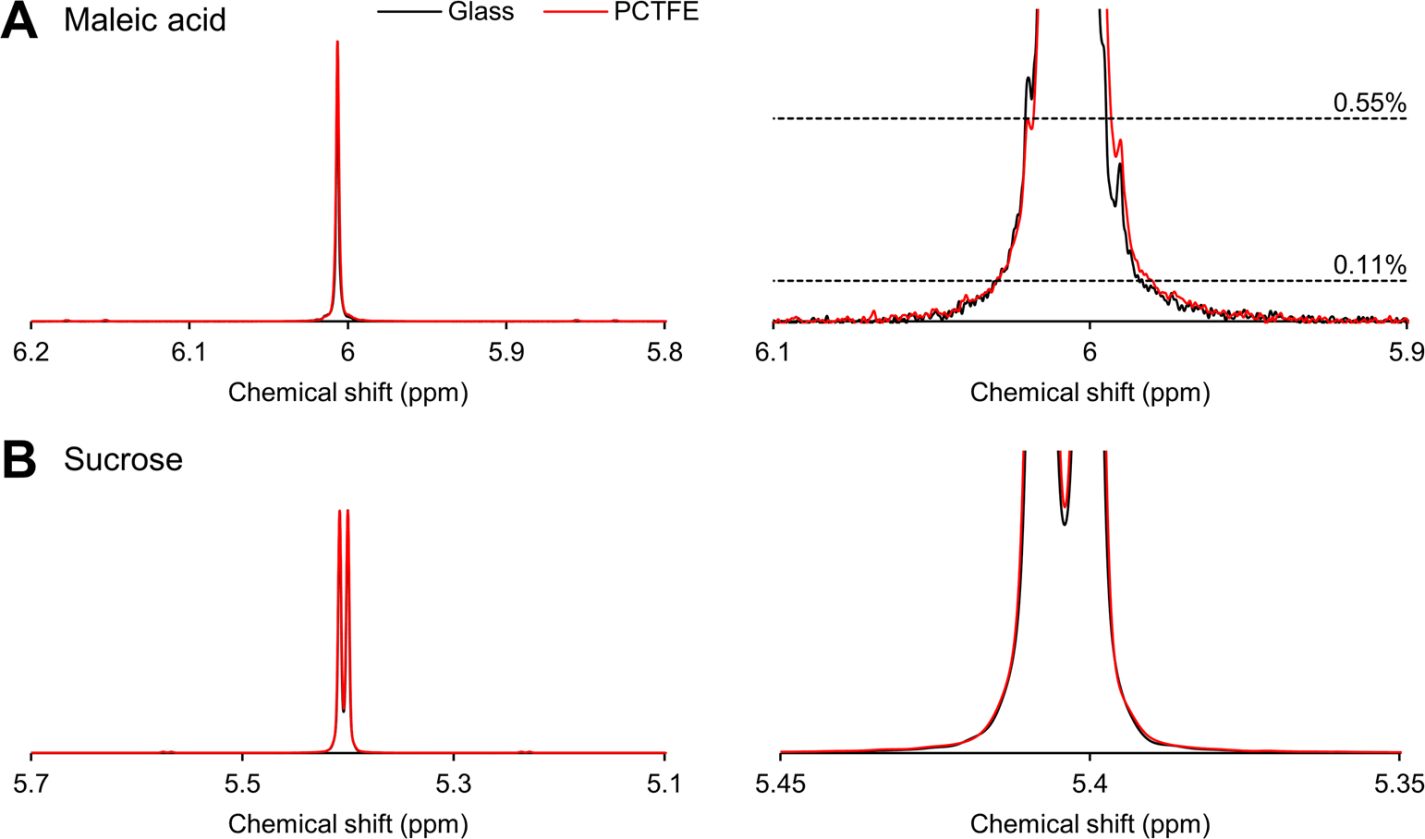
Selected sections of 500 MHz ^1^H NMR with overlaid spectra of PCTFE flow cell (red) and glass flow cell (black) of (A) maleic acid (δ_H_ = 6.02 ppm) and zoom-in; (B) sucrose doublet (δ_H_ = 5.4 ppm) and zoom-in. All peaks are referenced to the resonance of DSS-d_6_ at 0 ppm.

System performance was evaluated with intercalating injections of sucrose and blanks, and citric and maleic acid, and compared to the BEST-NMR system. Selected spectra are presented in Figure 3 and the results are summarized in Table 1. Line width, line shape, and line splitting are important benchmarks for the spectral quality of a flow cell. A broad line width can indicate inhomogeneities in the magnetic field caused by for example sample heterogeneity,^49^ misalignment of the flow cell,^49^ heterogeneity of the flow cell material or surfaces,^26^ or magnetic susceptibility mismatch near the coil.^39^ Similarly, a high line splitting percentage indicates a low resolving power between two spins, *ergo*, low resolution. Spectra of the singlet of maleic acid (Figure 3A) for the PCTFE and glass flow cells are near identical. Likewise, the sucrose doublet in Figure 3B shows that the resolution of the novel PCTFE flow cell at 27.9±1% is nearly equal to that of the glass reference cell at 25.2±2.7%. A quantitative comparison of the peak characteristics of sucrose, citric acid, and maleic acid in Table 1 supports this observation. Although the peak width is marginally smaller for the glass flow cell, the deviation between samples is smaller for the PCTFE cell. These results show that the manufacturing process led to fluoropolymer flow cells with high-quality surfaces that produce spectra on par with glass flow cells. We attribute this to the effect of compression molding to smooth the flow cell inner surfaces. In earlier generations of the PCTFE cells fabricated by machining methods only, the spectral quality was significantly lower than in the glass cells (details in Table S1 and Figure S3).

**Table 1:** Peak Width at Indicated Peak Heights and Relative Standard Deviation (RSD) of Peak Area for Selected Peaks per Sample

sample	δ_H_ (ppm)	flow cell	peak width (Hz; means ± SD) at peak height (%) (*n* = 48)	RSD peak area (%)
sucrose	3.66	PCTFE	1.90 ± 0.04 (50%)	0.35
		glass	1.82 ± 0.1 (50%)	0.75
citrate	2.59	PCTFE	1.35 ± 0.05 (50%)	0.12
		glass	1.39 ± 0.1 (50%)	0.84
maleic acid	6.02	PCTFE	31.75 ± 0.50 (0.11%)	0.53
			16.63 ± 0.27 (0.55%)	
			1.27 ± 0.05 (50%)	
		glass	31.80 ± 2.0 (0.11%)	0.96
			16.17 ± 0.9 (0.55%)	
			1.19 ± 0.1 (50%)	

The low relative standard deviation in peak area demonstrates the repeatability and robustness of the designed platform. The repeatability of the new platform is slightly better than that of the BEST-NMR reference, despite using a smaller fill factor. This can be attributed to repeatable positioning of the sample and magnetic susceptibility matching of the materials used.^39^ The sample-to-sample carryover was determined between intercalating injections of sucrose samples and blanks. For the ^1^H signals at 3.66 and 5.4 ppm, less than 0.2% of the original peak area was measured in the blank. Similar values were found with the BEST-NMR system; however, this system employed wash slugs of deuterated solvent. In comparison, Kautz et al. reported a carryover of 5% without wash segments in their fluorinated SFA-NMR system, which was attributed to a residual fused silica segment.^3^ With two subsequent blanks, no carryover could be detected in the second blank anymore. This indicates that carryover can be practically eliminated if desired.

### Sample Preconditioning During Transfer

**Figure 4:**
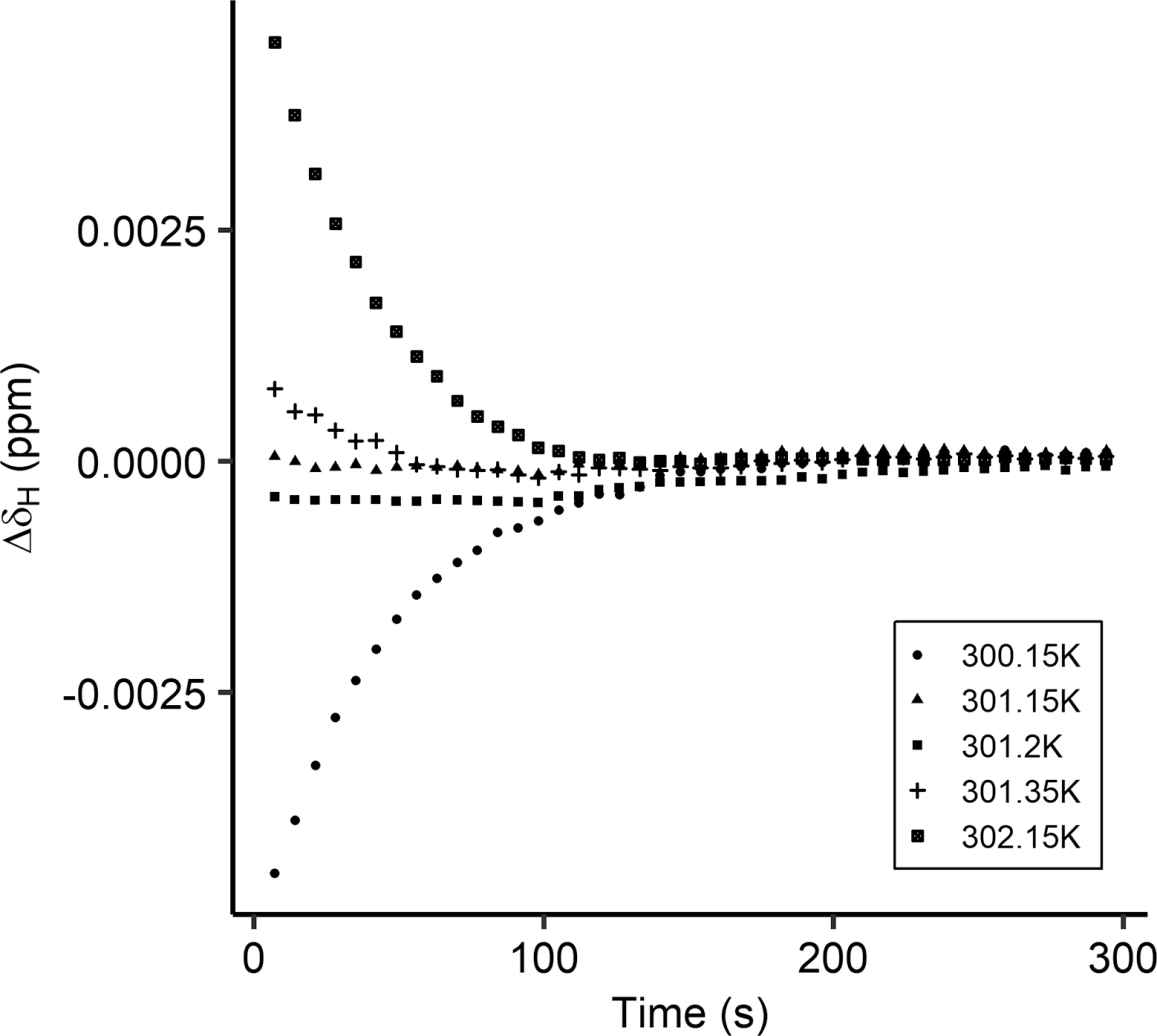
Delta chemical shift of maleic acid, relative to its final shift at δ_H_ = 6.31918 ppm, for various water bath temperatures, NMR probe temperature of 301.2 K, and ambient temperature of approximately 293.1 K.

In NMR analysis, it is essential to equilibrate the sample temperature prior to analysis, as a drift in sample temperature during acquisition will cause a drift in chemical shift. The umbilical accessory allows to precondition the sample temperature up to the NMR probe to reduce equilibration time inside the probe and increase sample throughput. To evaluate this effect, the drift in chemical shift of maleic acid was measured over a period of 300 at 7 s intervals for various water bath temperatures, probe temperature of 301.2 K, and a steady ambient temperature of 293.1 K. Figure 4 shows a clear optimum water temperature of 301.15 K, at which the drift is negligible. However, deviating by only 1 K of the optimum, the equilibration time increases to 120 s. The water bath temperature is not an absolute calibrated value, and the optimum needs to be finetuned for specific environmental and transfer conditions. These results demonstrate that a well-tuned system can achieve a drastic timesaving on the sample cycle time without affecting the spectral quality.

### Automated Screening of Urine

**Figure 5:**
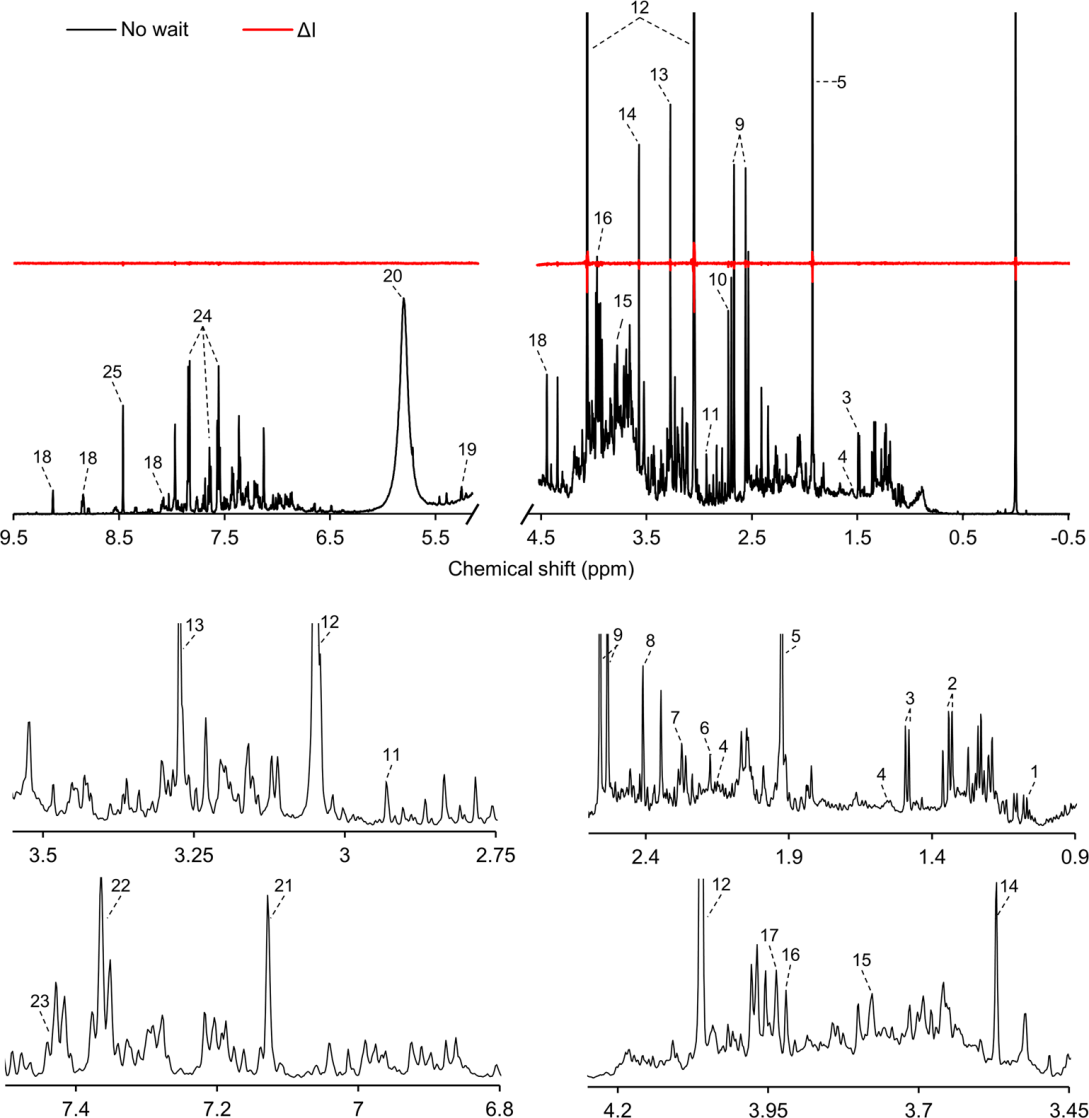
Typical 600 MHz ^1^H NMR spectra of human urine using PCTFE flow cell without in-probe temperature equilibration (black) and a delta-line compared to 300 s. equilibration time (red). Tentative peak annotations: 1. methylsuccinic acid (d, 1.07 ppm); 2. l-threonine (d, 1.33 ppm); 3. l-alanine (d, 1.49 ppm); 4. adipic acid (m, 1.55 ppm, m, 2.15 ppm); 5. acetic acid (s, 1.93 ppm); 6. acetone (s, 2.17 ppm); 7. acetoacetic acid (s, 2.27 ppm); 8. pyroglutamic acid (m, 2.41 ppm); 9. citric acid (d, 2.55 ppm, d, 2.68 ppm); 10. dimethylamine (s, 2.72 ppm); 11. trimethylamine (s, 2.93 ppm); 12. creatinine (s, 3.05 ppm, s, 4.06 ppm); 13. methanol (s, 3.27 ppm); 14. glycine (s, 3.57 ppm); 15. guanidoacetic acid (s, 3.78 ppm); 16. creatine (s, 3.92 ppm); 17. glycolic acid (s, 3.94 ppm); 18. trigonelline (s, 4.44 ppm); 19. d-glucose (d, 5.21 ppm); 20. urea (s, 5.80 ppm); 21. l-histidine (s, 7.13 ppm); 22. imidazole (s, 7.36 ppm); 23. mandelic acid (s, 7.44 ppm); 24. hippuric acid (m, 7.56 ppm, tt, 7.64 ppm, dd, 7.84 ppm); 25. formic acid (s, 8.46 ppm).

The applicability of the developed platform was demonstrated by acquiring spectra of urine from healthy volunteers and comparing spectra with and without temperature equilibration. Two spectra of the same sample were recorded consecutively without locking, tuning, and shimming before the acquisition. The first spectrum was acquired directly without temperature equilibration delay, and acquisition took approximately 300 s. The second spectrum was recorded directly thereafter, effectively resulting in at least 300 s of temperature equilibration. Figure 5 shows a typical spectra acquired without temperature equilibration in the probe, with tentative peak annotation of the typical metabolites found in urine.^50^ The delta line shows negligible difference in peak intensity between spectra acquired without temperature equilibration and after a 300 s delay. This confirms the previous result that with a well-tuned thermostated transfer line, the sample temperature is sufficiently equilibrated during the transfer. As a result, the sample cycle time is halved and throughput is doubled. This reduction is especially important for rapid screenings with short acquisition times of 30–40 s, where a temperature equilibration of 120 s would otherwise still be necessary, as demonstrated above.

Carryover of the spiked compounds was assessed from control urine to blanks, and from spiked urine to blanks, where samples were measured with 32 scans and blanks with 64 scans. All peaks were normalized to TSP by peak area. Carryover to the blank was ≤0.65% for all compounds, and no carryover was detected in the subsequent blank. Carryover can be further eliminated by adding an additional wash segment between samples, depending on the application. The carryover with urine is slightly higher than with standard samples, which may be explained by the sample complexity and the presence of more hydrophobic compounds. These results are an improvement over reported carryover with the BEST-NMR system for urine^4^ and other complex biological samples.^34^ This demonstrates that the system is reliable for analysis of complex samples.

## 4 Conclusion

This work successfully demonstrated an automated, segmented-flow analysis – NMR platform, including a novel PCTFE fluoropolymer flow cell. PCTFE proved an excellent material and enabled manufacturing of an intricate flow cell design while achieving high-quality inner surfaces with thermal compression molding. Spectral performance of the novel flow cell equals that of commercial glass flow cells. A fully automated workflow was established between the autosampler, auxiliary hardware, and spectrometer, and integrated readily with the spectrometer ecosystem. The PCTFE flow cell was connected to a commercial coaxial heat exchanger accessory to precondition the sample during transfer. It was shown that after tuning of the heat exchanger, temperature equilibration in the probe can be reduced to a minimum, hence increasing the system throughput. The platform was able to robustly transfer samples to the magnet, with better repeatability of peak area than the commercial benchmark, and is now in use for routine analysis of 25,000-50,000 samples per year. The fully fluorinated flow path in combination with fluorinated oil provided low carryover for both standard and urinary samples without the use of additional wash steps. We envision that this fluorinated SFA-NMR platform can open a new route to fast, robust, and high-quality flow NMR for (industrial) screening studies.

## Supporting Information Available

^1^H NMR background spectra of FC-72 oil and PCTFE material, and spectral evaluation of an earlier generation of a PCTFE flow cell compared to a commercial glass flow cell (PDF)
Detailed technical drawing of the PCTFE flow cell (PDF)
