# Supplementary material for: Automated Segmented-Flow Analysis – NMR with a Novel Fluoropolymer Flow Cell for High-Throughput Screening
Source: Anal Chem. 2022 Oct 27;94(44):15350–8. doi: 10.1021/acs.analchem.2c03038 (PMC9647699; doi:10.1021/acs.analchem.2c03038)
Supplement: Supplementary file 1 — ac2c03038_si_004.pdf [file ac2c03038_si_004.pdf]

Supplementary Information

Automated segmented-flow analysis – NMR with a novel fluoropolymer flow cell for high-throughput screening

Bert Wouters[a‡*], Paul Miggiels[a‡], Roland Bezemer[b], Elwin A.W. van der Cruijsen[b], Erik van Leeuwen[b], John Gauvin[b], Klaartje Houben[b], Karthick Babu Sai Sankar Gupta[c], Paul Zuijdwijk[b], Amy Harms[a], Adriana Carvalho de Souza[b], and Thomas Hankemeier[a*]

[a] Metabolomics and Analytics Centre, Leiden Academic Centre for Drug Research, Leiden University, Einsteinweg 55, 2333 CC Leiden, The Netherlands

[b] DSM Biotechnology Center, Alexander Fleminglaan 1, 2613 AX Delft, The Netherlands

[c] Leiden Institute of Chemistry, Leiden University, Einsteinweg 55, 2333 CC Leiden, The Netherlands

(‡) Equal contribution

(*) corresponding author

Einsteinweg 55, 2333 CC Leiden, The Netherlands

Tel.: +31 71 527 4226, E-mail: hankemeier@lacdr.leidenuniv.nl

Table of content

Figure S1	1H NMR spectrum of FC-72 fluorinated oil
Figure S2	1H NMR spectrum of D_2O in PCTFE flow cell
Figure S3	Selected spectra of 1st generation PCTFE and glass flow cell
Table S1	Characterization of selected peaks of 1st generation PCTFE and glass flow cell

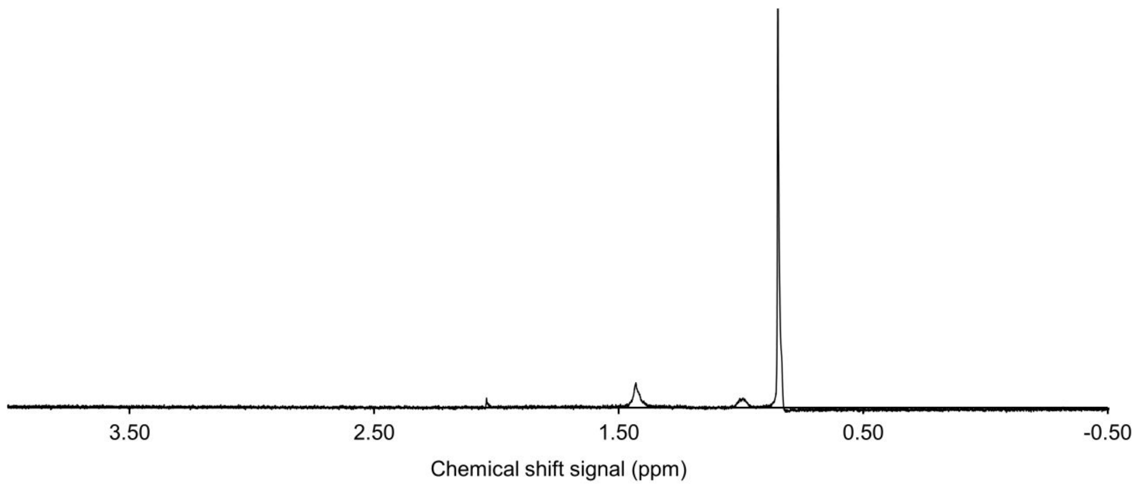

Figure S1. 600 MHz 1H NMR spectra of FC-72 fluorinated oil. Spectrum zoomed into the region of δ_H=-0.50-4.00 ppm. Residual proton signal at δ_H=0.80 ppm indicate presence of impurities in the oil. Spectra recorded in a 3.0 mm glass NMR tube on Bruker UltraShield 600 MHz with Avance III HD spectrometer and cryoprobe with the following parameters: pulse program (pulprog): zg; water suppression power level: n/a; number of scans (ns): 16; relaxation delay (d1): 5 s; acquisition time (aq): 2.04 s; pulse length (p1): 8 μs; receiver gain: 16; probe temperature: 300 K; no lock solvent used.

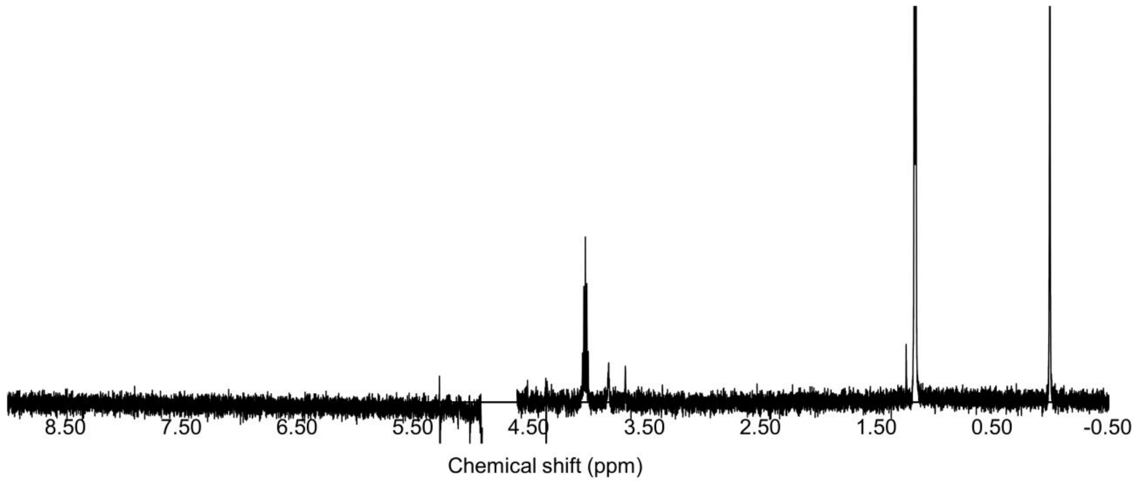

Figure S2. 1H NMR spectrum of D_2O in PCTFE flow cell. All peaks are references to the resonance of DSS at 0 ppm. Spectra recorded on a Bruker Avance III HD 500 MHz Ultrashield spectrometer with helium-cooled cryoprobe installed. Acquisition parameters: 90° pulse calibration and shimming prior to each sequence; locking, automatic tuning & matching; 120 s equilibration delay; probe temperature: 298 K; pulse program (pulprog): zgcppr; water suppression: 70 dB/5Hz; number of scans (ns): 32; relaxation delay (d1): 4.0 s; acquisition time (aq): 3.12 s; pulse length (p1): 7 µs; receiver gain: 32; spectral width (sw): 21.0028 ppm.

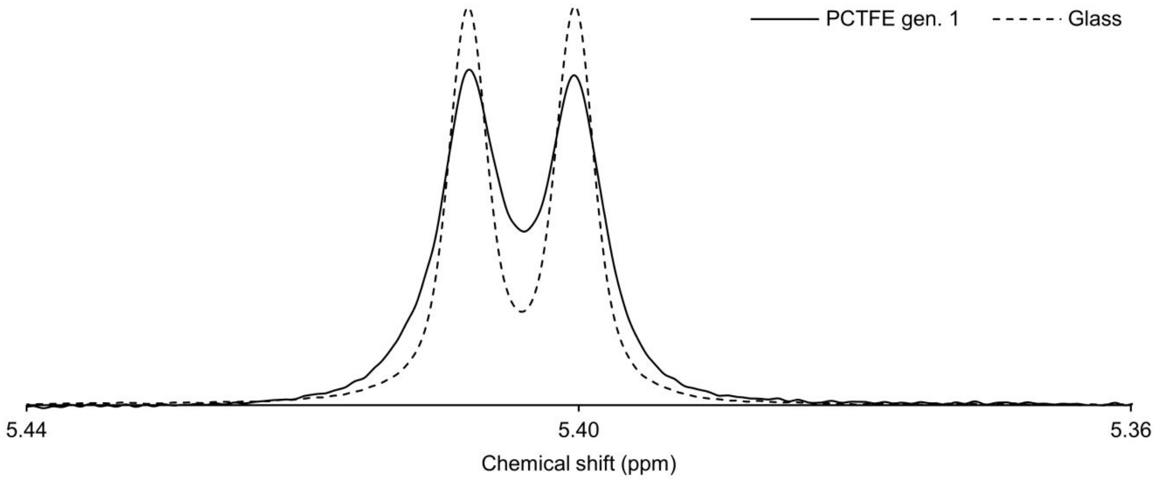

Figure S3. **1H NMR spectra of sucrose in D_2O (2 mM) at 5.40 ppm of first generation PCTFE flow cell (solid) and commercial glass flow cell (dotted).** The first generation PCTFE cell, manufactured without thermal compression moulding, shows broader peaks and

lower resolution than the commercial glass cell. All peaks are referenced to the resonance of DSS at 0 ppm. Spectra were recorded with the SFA-NMR platform (PCTFE) or BEST-NMR platform (glass) coupled to a Bruker Avance III HD 500 MHz Ultrashield spectrometer with a helium-cooled cryoprobe installed. Acquisition parameters as in Figure S2.

Table S1. Peak characterization of selected peaks for 1st generation PCTFE flow cell and commercial glass flow cell. The 1st generation PCTFE flow cell, manufactured without thermal compression moulding, still has lower spectral quality than the commercial glass flow cell. Acquisition details are described in Figure S3.

Sample	δ_H (ppm)	Flow cell material	Peak width in Hz (mean±SD) at peak height (%) (n=48)
5 mM sucrose	3.66	Glass	1.7±0.0 (50%)
		PCTFE (1st gen.)	3.0±0.1 (50%)
17 mM Maleic acid	6.02	Glass	1.2±0.1 (50%)<br>16.2±0.9 (0.55%)<br>31.8±2.1 (0.11%)
		PCTFE (1st gen.)	2.1±0.3 (50%)<br>21.5±0.9 (0.55%)<br>40.2±2.4 (0.11%)
17 mM Citrate	2.59	Glass	1.4±0.1 (50%)
		PCTFE (1st gen.)	2.2±0.2 (50%)
