# Supplementary material for: Automated Segmented-Flow Analysis – NMR with a Novel Fluoropolymer Flow Cell for High-Throughput Screening
Source: Anal Chem. 2022 Oct 27;94(44):15350–8. doi: 10.1021/acs.analchem.2c03038 (PMC9647699; doi:10.1021/acs.analchem.2c03038)
Supplement: Supplementary file 2 — ac2c03038_si_005.pdf [file ac2c03038_si_005.pdf]

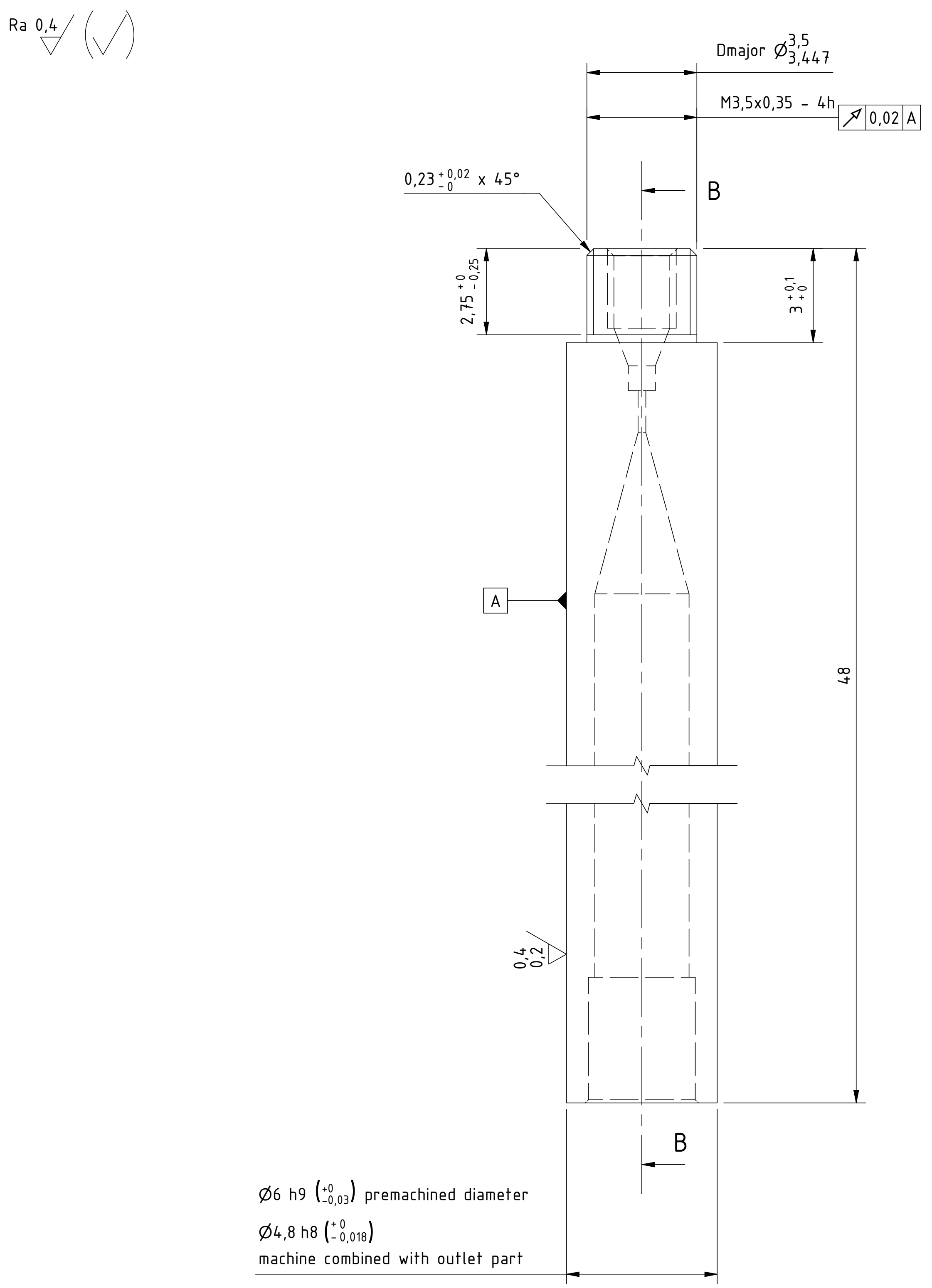

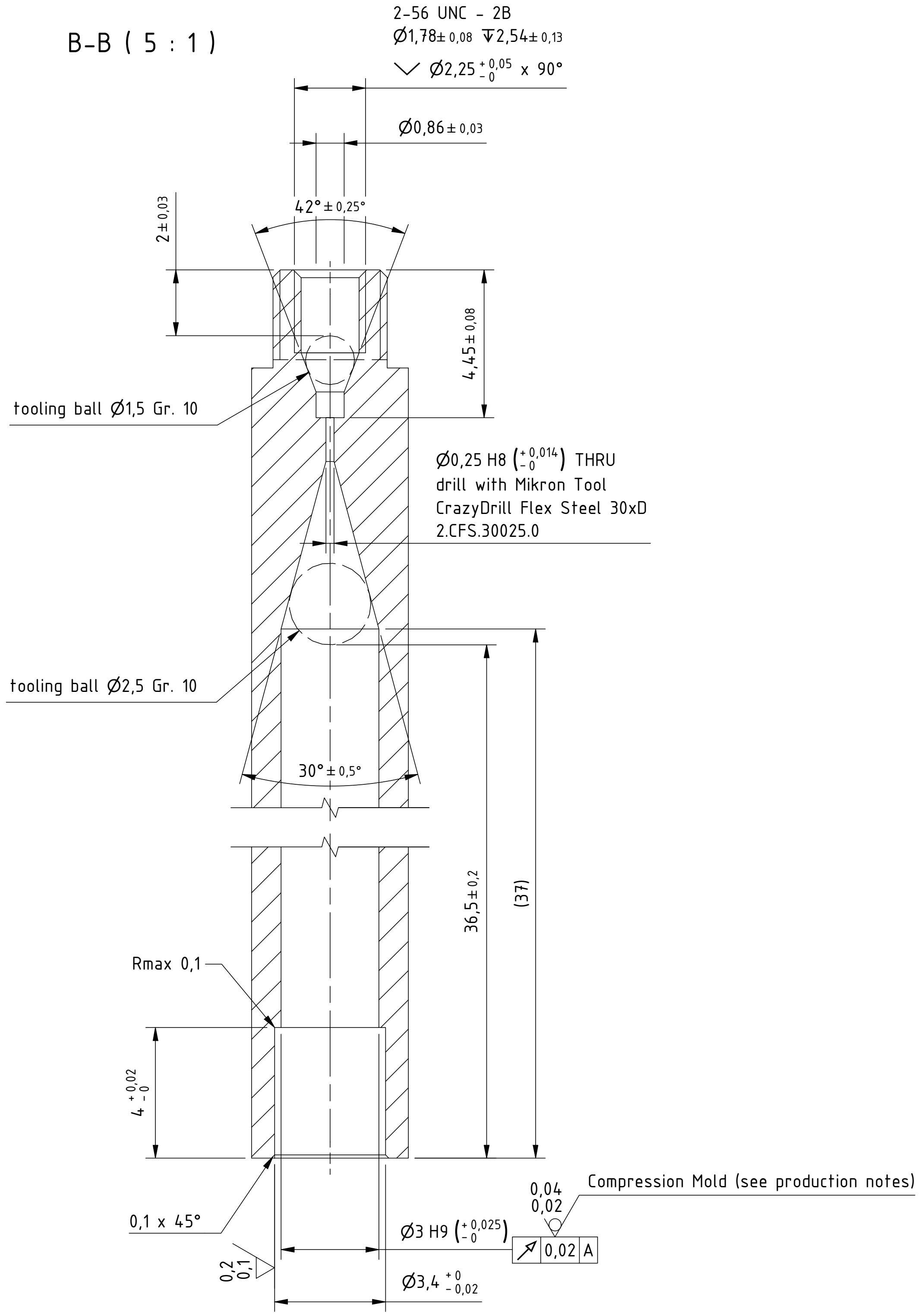

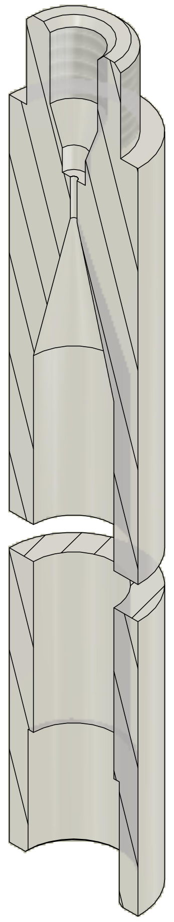

**Dimensional tolerancing:** NEN-ISO 2768-fH		**Geometrical tolerancing:** NEN-ISO 1101	**Surface roughness:** NEN-EN-ISO 1302	**Material:** PCTFE (Kel-F®)
**Designed by:** RCT Zwier	**Design status:** Released	**Release date:** 20-5-2021	**Group:** ABS\SPB	3th angle · LEIDEN UNIVERSITY PROPRIETARY THIS DOCUMENT CONTAINS CONFIDENTIAL PROPRIETARY INFORMATION THAT IS PROPERTY OF LEIDEN UNIVERSITY DO NOT DISCLOSE TO OR DUPLICATE FOR OTHERS EXCEPT AS AUTHORIZED BY LEIDEN UNIVERSITY
Leiden university · Leiden institute of physics Fine mechanical department		**Partname:** Flowcell 6.0 ID=3mm		**Qty:**
Niels Bohrweg 2 2333 CA Leiden Netherlands · FMD		**Project:** HT-NMRFlowcell		**Scale:** 5 : 1 · **Units:** mm · A3 · **Sheet:** 1 /2
		**Filepath:** R:\2005 LACDR projecten\LACDR-SPB-ABS-Hankemeier\2019-RZ-HT NMR Flowcell Gen 5 (Bert Wouters)\Flowcell 6.0\Flowcell 6.0 ID=3mm.ipt		

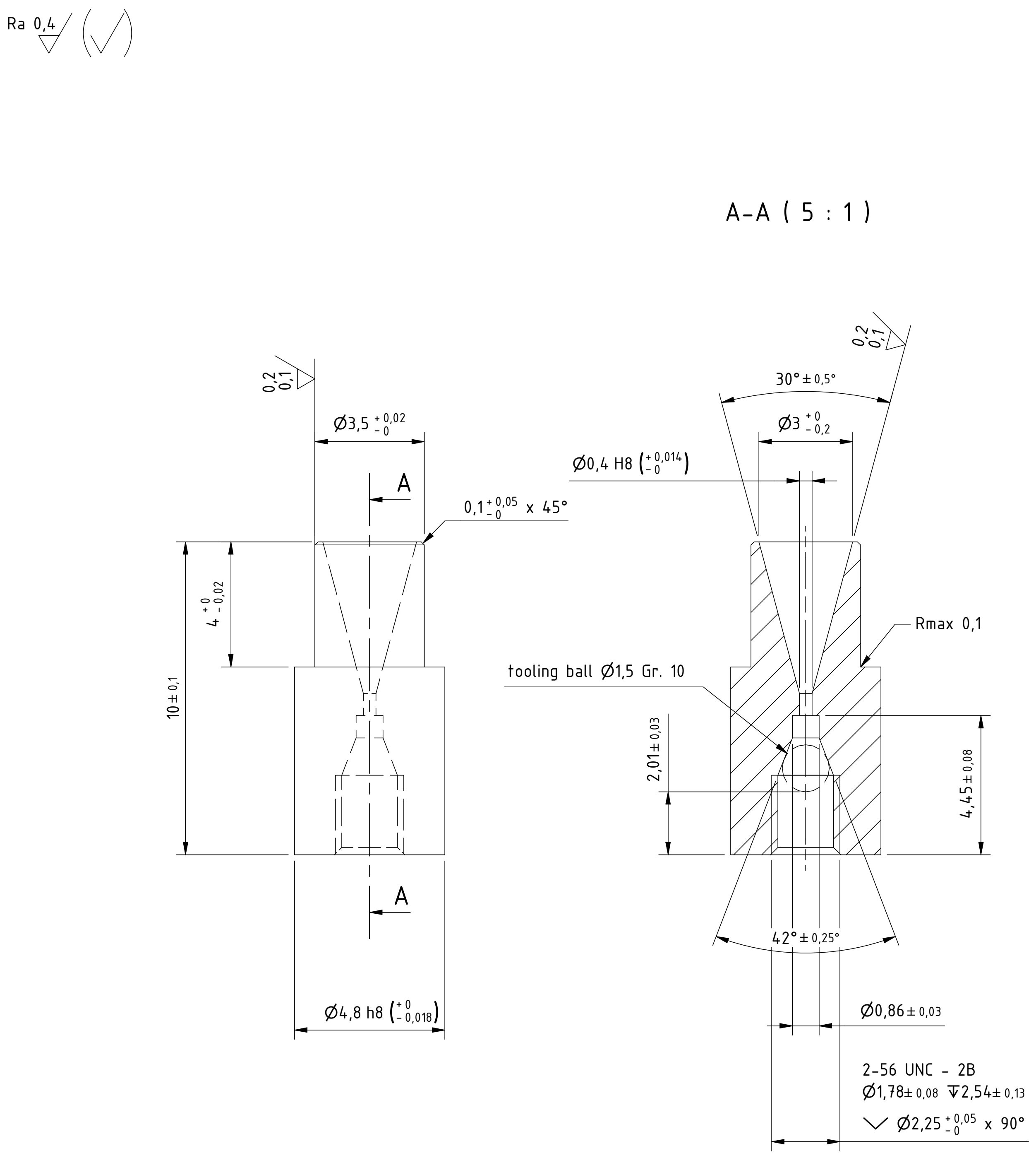

**Dimensional tolerancing:** NEN-ISO 2768-fH		**Geometrical tolerancing:** NEN-ISO 1101	**Surface roughness:** NEN-EN-ISO 1302	**Material:** PCTFE (Kel-F®)	
**Designed by:** RCT Zwier	**Design status:** Released	**Release date:** 20-5-2021	**Group:** ABS/SPB	3th angle	LEIDEN UNIVERSITY PROPRIETARY THIS DOCUMENT CONTAINS CONFIDENTIAL PROPRIETARY INFORMATION THAT IS PROPERTY OF LEIDEN UNIVERSITY DO NOT DISCLOSE TO OR DUPLICATE FOR OTHERS EXCEPT AS AUTHORIZED BY LEIDEN UNIVERSITY
Leiden university<br>Leiden institute of physics<br>Fine mechanical department<br>Niels Bohrweg 2<br>2333 CA Leiden<br>Netherlands<br>FMD		**Partname:** Flowcell 6.0 Outlet		**Qty:**	
		**Project:** HT-NMR Flowcell		**Scale:** 5 : 1	**Units:** mm · A4 · **Sheet:** 2 /2
		**Filepath:** R:\2005 LACDR projecten\LACDR-SPB-ABS-Hankemeier\2019-RZ-HT NMR Flowcell Gen 5 (Bert Wouters)\Flowcell 6.0\Flowcell 6.0 Outlet.ipt			
